# Direct Thermal Growth of Large Scale Cl-doped CdTe Film for Low Voltage High Resolution X-ray Image Sensor

**DOI:** 10.1038/s41598-018-33240-1

**Published:** 2018-10-04

**Authors:** Silah Lee, Jin Sung Kim, Kyeong Rok Ko, Gun Hwan Lee, Dong Jin Lee, Dong wook Kim, Jin Eui Kim, Ho Kyung Kim, Dong Woon Kim, Seongil Im

**Affiliations:** 1 Department of Physics and Institute of Physics and Applied Physics, Yonsei University, 50 Yonsei-ro, Seodaemun-gu Seoul, 120-749 Korea; 2 Department Of Surface Technology, Korea Institute of Materials Science (KIMS), 797 Changwon-daero, Seongsan-gu, Changwon-si, Gyeongsangnam-do 51508 Korea; 3 Rayence Co.,Ltd, 14, Samsung 1-ro 1-gil, Hwaseong-si, Gyeonggi-do 18449 Korea; 4 School of Mechanical Engineering, Pusan National University, 2, Busandaehak-ro 63beon-gil, Geumjeong-gu, Busan 46241 Korea

## Abstract

Polycrystalline cadmium telluride (CdTe) X-ray photodetector with advanced performance was fabricated in a Schottky diode form by direct thermal deposition (evaporation) on pixelized complementary metal oxide semiconductor (CMOS) readout panel. Our CdTe X-ray detector shows such a variety of benefits as relatively low process temperature, low cost, low operation voltage less than 40 V, and higher sensitivity and spatial resolution than those of commercial a-Se detectors. CdTe has cubic Zinc Blende structure and maintains p-type conduction after growth in general. For low voltage operation, we succeeded in Cl doping at all stage of CdTe film deposition, and as a result, hole concentration of p-type CdTe was reduced to ~10^12^ cm^−3^ from ~10^15^ cm^−3^, and such concentration reduction could enable our Schottky diode with Ti electrode to operate at a reverse bias of less than 40 V. Our CdTe Schottky diode/CMOS pixel array as a direct conversion type imager demonstrates much higher resolution X-ray imaging in 7 × 9 cm^2^ large scale than that of CsI/CMOS array, an indirect conversion imager. To our limited knowledge, our results on polycrystalline CdTe Schottky diode/CMOS array would be very novel as a first demonstration of active pixel sensor system equipped with directly deposited large scale X-ray detector.

## Introduction

Until recent years, many types of active matrix flat panel X-ray imager have been developed since back panel transistors began to support front panel X-ray detectors^1–25^. While back panel thin-film transistors (TFTs) play as driving and switching elements for large scale active X-ray imager^1–3,10^, complementary metal oxide semiconductor (CMOS) transistors on Si wafer have been used for small area but high resolution image. Medical X-ray imaging applications such as radiography^4,10,15,16,19^, mammography^5,6,17–19^, fluoroscopy^7,10^, angiography^20^ and radiotherapy have thus been developed with flat panel X-ray image sensor. X-ray image sensor pixels are categorized into two types: indirect and direct conversion types according to front panel X-ray detection principle. In the indirect type, energetic X-rays excite scintillator such as cesium iodide (CsI), to be converted to visible light^1–3,18,20^. Those visible photons are recieved by an array of amorphous silicon photodiodes located below the scintillator resulting in electron-hole carriers, so that a back panel device (TFT or CMOS) transports the signal charge carriers to external readout integrated circuit (IC). In contrast, the direct type uses photoconductor materials to directly convert X-ray photons to electrical signals^5–11,13–15,17,19^. Although indirect conversion possibly utilizes the detection capability of high performance photodiode, it shows inherent drawbacks such as complexity of p-i-n photodiode fabrication and spatial resolution limit due to light spreading of scintillator^26^. Hence, the direct conversion device with superior spatial resolution and improved signal-to-noise ratio (SNR) has attracted much attention from researchers^8,9^.

Commercial direct type active matix X-ray imager consists of amorphous selenium (a-Se) photodetector (or photoconductor) and a-Si TFT switch in sensor system; the a-Se detector/a-Si TFT combination is classified as passive pixel sensor (PPS) which is different from active pixel sensor (APS) using Si-CMOS device. Direct type APS system is more desirable than PPS system because of its more highly-resolved quality in static and even dynamic images^21–25^. However, any large area APS for direct type detection has rarely been reported yet, because several of difficult issues remain to be resolved: selecting a proper photoconductor material, lowering process costs, forming the photoconductor/CMOS interface without damage, and lowering detector operation voltage. First, in terms of photoconductor materials selection, commercial a-Se, cadmium telluride (CdTe), lead iodide (PbI_2_), and lead oxide (PbO), and mercury iodide (HgI_2_) have been mentioned as shown in Supplementary Table S1 ^9,11,13^. Commercial a-Se should be as thick as a few hundred μm for somewhat guaranteed stability, requiring at least a few hundred volts for detector operation. In general, a-Se is not matched to APS applications. According to Table S1, CdTe seems to be the most process-stable during thermal deposition because others show much lower process temperature, which means that they are vulnerable to elevated temperature. Next, detector/CMOS interfacing process may be the most important and difficult part for APS system, because such detector (or photoconductor) layer should be directly deposited on pixelized CMOS structures. For such detector deposition (or detector alignment) on CMOS pixel, two methods have been attempted so far: direct thermal deposition and pixel-to-pixel direct bumping. Direct thermal deposition on pixelized CMOS structure can damage the CMOS itself if the deposition temperature is too high, while direct pixel-to-pixel bumping requires very precise alignment between photoconductor and CMOS pixels. For direct thermal deposition of photoconductor, *in-situ* chemical doping would be very difficult to be quantitatively controlled, however is worthwhile to study further in the near future. Information in Supplementary Fig. S1 summarizes and categorizes all abovementioned X-ray imaging systems including front and back panel details.

In the present study, we selected polycrystalline cadmium telluride (CdTe) film as an advanced photoconductive material, expecting several benefits over commercial detector materials (e.g. a-Se): decent process temperature, low cost process, low operation voltage less than 40 V, high X-ray sensitivity, and ambient/or process stablity. More than that, large scale direct thermal deposition (evaporation) is technically possible on pixelized CMOS structure. CdTe has cubic Zinc Blende structure and maintains p-type conduction after growth in general^27,28^. For low voltage operation, we applied Cl doping at all stages of CdTe film deposition to control or reduce the hole concentration of p-type CdTe, which then plays as Schottky diode material for Ti(or Al) electrode showing ohmic behavior for Au^29–32^. As a result, our CdTe Schottky diode/CMOS array demonstrates much higher resolution X-ray imaging in 7 × 9 cm^2^ large scale as a direct conversion APS imager than that of CsI/CMOS array, an indirect conversion imager. To our limited knowledge, our results on polycrystalline CdTe Schottky diode/CMOS array would be very novel as a first demonstration of APS system equipped with directly deposited large scale X-ray detector. (According to Table S1, overall properties such as electron-hole pair creation energy (W) and mobility × carrier life time product (μτ) in our polycrystalline CdTe material are shown and regarded quite desirable).

## Result and Discussion

**Figure 1:**
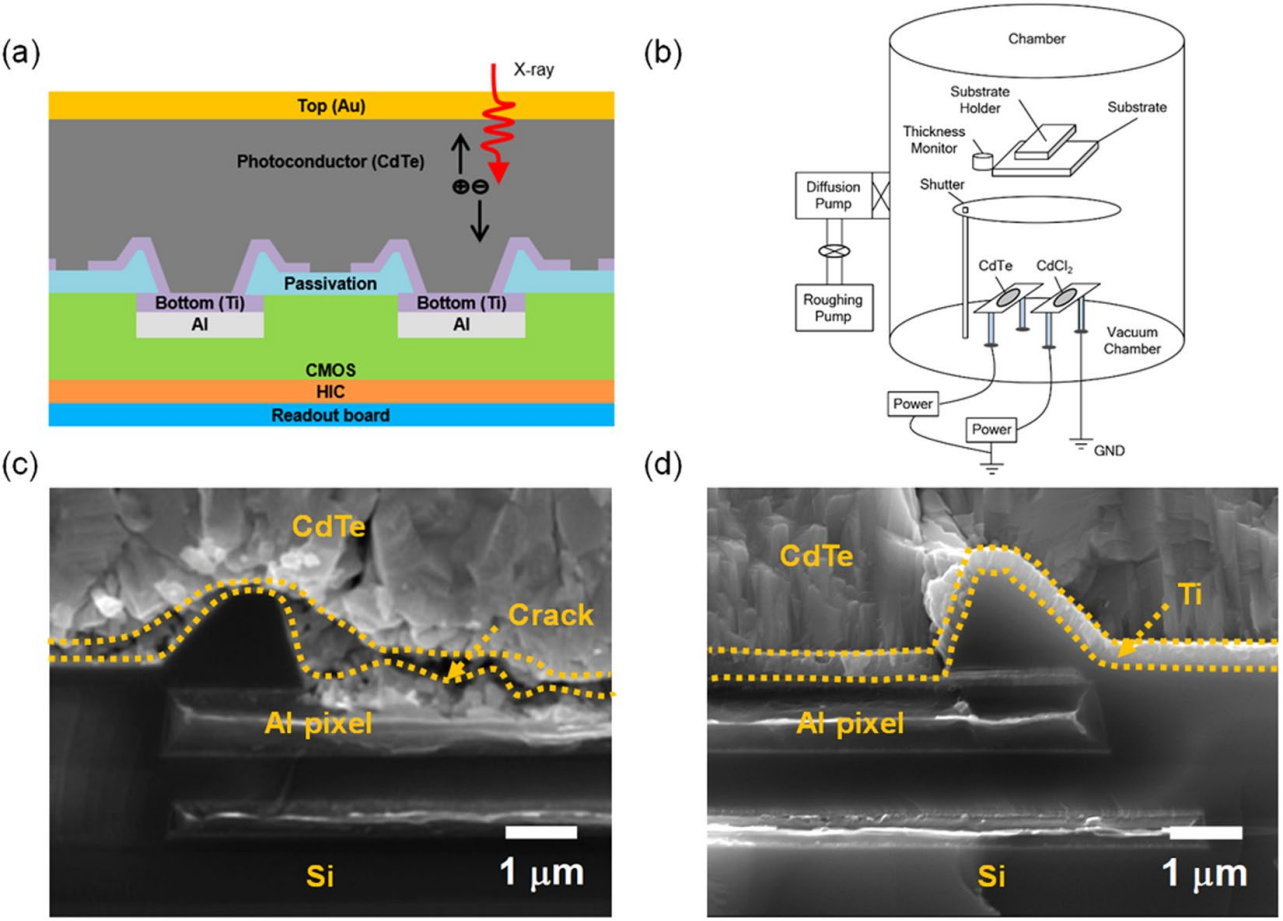
(**a**) A schematic cross section of p-type CdTe Schottky diode photodetector. Irradiated X-ray generates electron-hole pairs to be received by electrodes, and electrons are detected as signals by Ti Schottky electrode which is pixel-patterned for CMOS panel. (**b**) The evaporation chamber system with two source (CdTe and CdCl_2_ powders) boats which are to deposit CdTe film and simultaneously to dope Cl atoms. (**c**) Cross sectional scanning electron microscopy (SEM) images of CdTe films on Al (**d**) and Ti pixel electrodes. Cracks and delamination were observed at the Al/CdTe interface, while those damages were avoided using Ti film deposition on Al pixel.

Figure 1a shows a schematic cross section of our p-type CdTe Schottky diode photodetector directly prepared on CMOS pixels, which have patterned Ti electrodes to receive photo-carriers (photoelectrons) generated by X-ray irradiation. Individual Ti electrodes are separated by passivation layer of SiO_2_. For the top ohmic contact electrode on p-type CdTe, Au has been used. Details of CMOS readout circuits to support our APS system are seen in Supplementary Fig. S2. Figure 1b is the evaporation chamber system with two source boats which are to deposit CdTe film and simultaneously to dope Cl atoms. For Cl doping, we used CdCl_2_ source powders. Figure 1c,d display cross sectional scanning electron microscopy (SEM) images of CdTe films on Al and Ti pixel electrodes, respectively (pixel pattern device area = 20 × 20 mm^2^). Those two metal electrodes are supposed to form Schottky junction with p-type CdTe because their work functions are similar each other (~4.2 eV) but quite smaller than that of p-type CdTe (~5 eV)^33^. However, Al has very different thermal expansion properties from CdTe’s, finally causing interface cracking during the CdTe growth at an elevated substrate temperature of 400 °C as a result shown in Fig. 1c ^34,35^. In contrast, CdTe film growth on Ti appears successful as seen in Fig. 1d where Ti film pattern is added on Al pixel electrode (we thus call this new pattern as Ti pixel electrode from now on). It has been observed in our study that CdTe film and Ti keep good continuous interface without any crack. It is certainly because thermal expansion coefficient of Ti metal is close to that of CdTe, so we here utilized Ti pixel electrode for a proper Schottky diode formation^36^.

**Figure 2:**
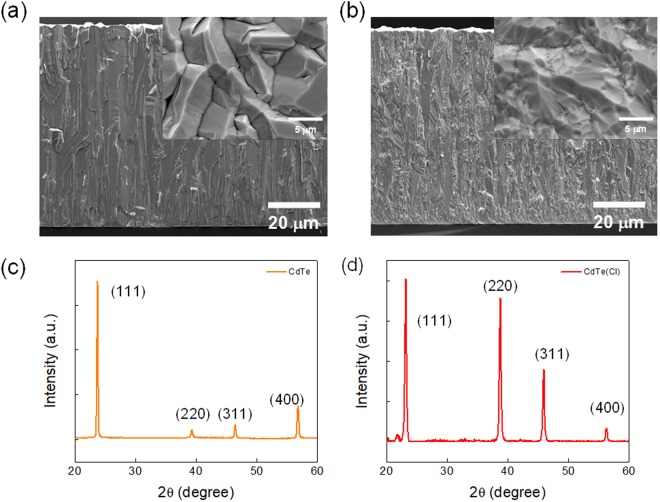
(**a**) The cross sectional SEM micrographs of CdTe films grown on Ti electrode at 400 °C, respectively without Cl (**b**) and with Cl doping. Each inset shows a magnified view. The X-ray diffraction (XRD) spectra from (**c**) un-doped CdTe (**d**) and Cl-doped CdTe films.

Figure 2a and b display the cross sectional SEM micrographs of our CdTe films grown on Ti electrode at 400 °C, respectively without and with Cl doping by CdCl_2_ co-evaporation (Evaporation details are in Experimental section). We deposited these semiconductor films on Ti-deposited 300 nm-thick SiO_2_/*p*-Si blank wafer without any pixel pattern, to investigate optimum deposition and doping conditions. Compared to the textured structure of Fig. 2a and its inset, the grains of Fig. 2b containing Cl dopants appear small and thin, much deviated from the texture form. It is highly suspected that Cl atoms might have controlled the growth of CdTe grain in size and morphology. Hence, the growth rate of Cl-doped CdTe film was, in fact, two times slower than that of undoped CdTe. The total thickness of our Schottky diode was observed to be ~150 μm after 4 hours of deposition without Cl doping as shown in SEM. But the same thickness was obtained in 8 hours with Cl doping. Along with the different SEM morphologies, the X-ray diffraction (XRD) spectra from CdTe without (Fig. 2c) and with Cl doping (Fig. 2d) also display noticeable difference each other. CdTe film without Cl dopant shows (111) orientation-dominated texture while such orientation becomes less strong but mixed with other orientations of (220) and (311) due to Cl doping. According to the literature, it is suspected that doped Cl atoms not only exist in the grain of CdTe film but also in the grain boundaries, so that they might retard the growth of CdTe crystals^30,31^.

**Figure 3:**
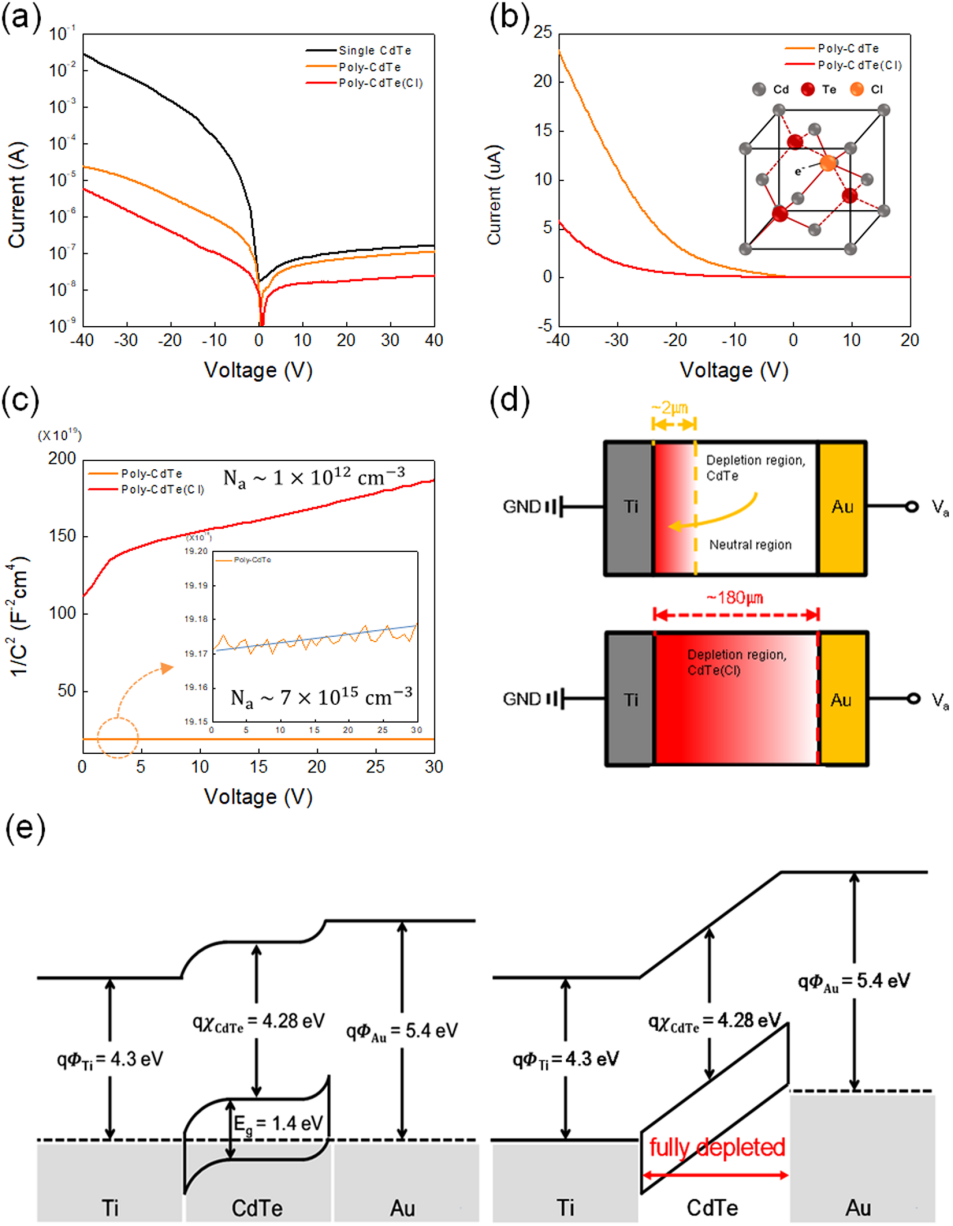
(**a**) The logarithmic and (**b**) linear scale current-voltage (I-V) plots of poly-CdTe with and without Cl doping. Inset shows a schematic unit cell describing Cl dopant as electron donor. (**c**) 1/C^2^-V curve plot to estimate the hole concentrations of Cl-doped p-CdTe and un-doped CdTe (inset is for magnified view). (**d**) The charge depletion thickness without and with Cl doping under a reverse bias of 30 V. (**e**) Energy band diagrams of Cl-doped Schottky diode under two bias states: 0 volt and full depletion condition bias.

The electrical properties of Ti/polycrystalline (poly-) CdTe Schottky diode are certainly changed by the Cl doping. According to the logarithmic and linear scale current-voltage (I-V) plots of Fig. 3a,b, our poly-CdTe Schottky diode with Cl shows an order of magnitude lower ON and OFF state current than that of the other one without doping, while as a reference single crystalline p-CdTe Schottky diode displays much higher ON current by ~four orders of magnitude than poly-CdTe devices (in fact, the 1 cm^2^ small scale reference p-CdTe crystal has been used for detector in direct APS imager). For image contrast and resolution, OFF state current should be always important and be as low as possible. In reports, some of the doped Cl atoms are segregated in the grain boundaries, so that they might probably block the current leakage path^30,31^. In this regard, our Cl-doped CdTe diode could have advantages over undoped ones. The most interesting, however, must be the results from capacitance-voltage (C-V) measurements in Fig. 3c, according to which the hole concentration of p-CdTe is tremendously reduced to only ~10^12^ cm^−3^ by Cl doping as estimated from the slope of 1/C^2^-V curve in Schottky diode. Following is the relationship between carrier concentration *N*_*a*_ and the slope^37^.

1 $$Slope=\frac{2}{q{\varepsilon }_{CdTe}{\varepsilon }_{0}{N}_{a}}$$

where *q* is electronic charge, *ε*_*Cdte*_ is the dieletric constant of CdTe, and *ε*_0_ is the permittivity of free space.

Without Cl doping, the hole concentration appears to be as high as ~7 × 10^15^ cm^−3^, which is three-to-four orders of magnitude higher than the case of Cl-doped CdTe (see the inset 1/C^2^-V plot.) After the film deposition with CdCl_2_ co-evaporation, the Cl content was analyzed by ion chromatography and found to be ~0.5 ppm. This number can be converted to ~5 × 10^15^ cm^−3^ if we consider that the atomic density of CdTe crystal should be ~ a few number x 10^22^ cm^−3^. In other words, doped Cl concentration is expected to be more than ~5 × 10^15^ cm^−3^. Although some of doped Cl atoms are located in grain boundaries, many Cl atoms would substitute Te atoms as shown in a schematic crystal lattice (inset of Fig. 3b), donating electrons whose concentration could be in the order of ~10^15^ cm^−3 ^^29–32^. As a result, those number electrons would compensate most of the holes in principle, depleting the charge carriers. Still, how to reproducibly and precisely control the Cl doping to result in such a low hole carrier concentration of ~10^12^ cm^−3^ remains, to be done in the future. This charge depletion or carrier density reduction by Cl doping leads to the benefit of low voltage operation in Schottky diode X-ray detector. For instance, the charge depletion thickness is estimated to be ~2 μm without Cl doping under a reverse bias of 30 V, while it would be extended to over 180 μm with Cl doping as shown in the illustrations of Fig. 3d. Hence, under 45 V of reverse bias, our 150 μm-thick CdTe Schottky diode should be under fully charge-depleted state, which is already very sensitive to energetic photons. Following equation shows the relationship between carrier concentration and the depletion thickness^37^.

2 $${x}_{d}=\sqrt{\frac{2{\varepsilon }_{CdTe}{\varepsilon }_{0}({\rm{\Phi }}-{V}_{a})}{q{N}_{a}}}$$

where *Φ* is the built-in potential (~0.9 eV) between p-CdTe and Ti electrode, and *V*_*a*_ is the applied voltage. Since all necessary constants for CdTe, Au, and Ti electrode are well known, we could clearly imagine energy band states under two different bias conditions as described in Fig. 3e: 0 volt bias and full depletion condition bias^33^.

**Figure 4:**
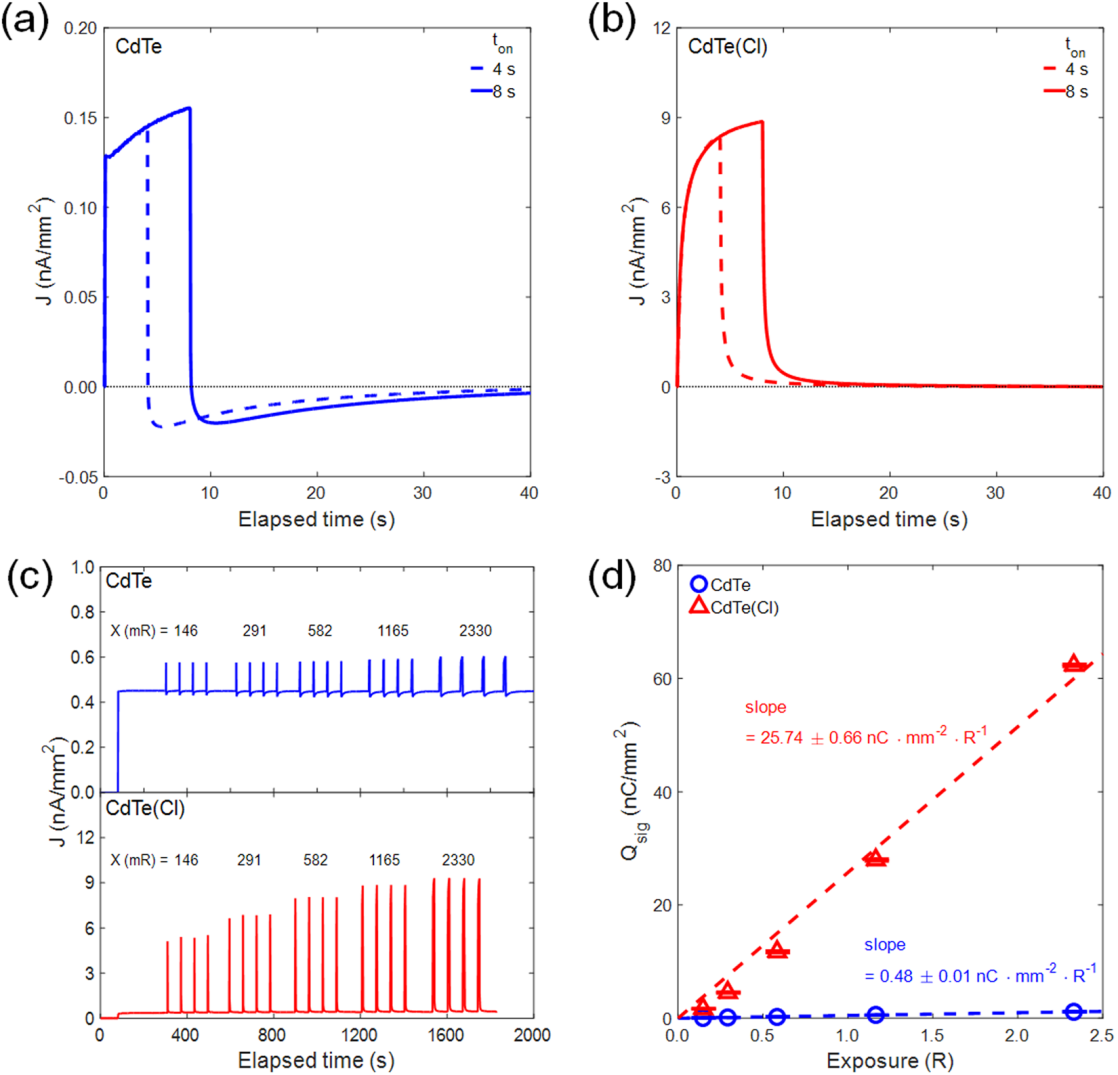
The X-ray-induced photocurrent as temporal response observed from Schottky diode (**a**) without and (**b**) with Cl doping. (**c**) The comparison of the temporal behavior of photocurrent signals from un-doped CdTe and Cl-doped CdTe devices under reverse bias. As soon as the bias is applied, a sudden jump of J is noted. (**d**) Signal charges are plotted as a function of the exposure level (R) which is measured by X-ray dosage meter, and R is proportional to peak acceleration voltage (90 kVp) x beam current (8 mA) x duration (8 sec). Sensitivity is defined as the slope, and it becomes 50 times higher by Cl doping.

In order to investigate any dynamic response of our 150 μm-thick CdTe Schottky diodes under 45 V reverse bias, we put the two Schottky diodes (with and without Cl doping) to the irradiation of energetic X-ray beam with 90 kVp and 8 mA for certain time durations (temporal exposure time, t_on_ = 4 and 8 s). In fact, these dynamic measurements are important in both respects of quantifying X-ray detector sensitivity and measuring X-ray ON/OFF response speed. Figure 4a shows X-ray-induced photocurrent as temporal response observed from the CdTe Schottky diode without Cl. According to results, its response time appears as fast as a few hundred microseconds but the signal height is as small as ~0.15 nA/mm^2^ (which is small enough to display even negative current density as a measurement error). However, under the same bias condition, our Cl-doped CdTe diode in Fig. 4b shows more than 50 times higher photocurrent signals, which reach to 9 nA/mm^2^ for 8 s duration. Response time behavior in Cl-doped CdTe device appears as slow as ~2 sec for 80% of complete rise and fall in signal, and it is possibly because larger area grain boundary might play as charge trap and de-trap site during device operation. Figure 4c compares the temporal behavior of photocurrent signals from un-doped and Cl-doped CdTe devices in a more general way. According to the temporally repeated response plots, X-ray-induced signals from Cl-doped CdTe are much larger than that from un-doped CdTe, and it is also noticed that the dark leakage current of both detectors abruptly increases at the early stage after the application of 45 V reverse bias. The average levels of dark current density (J) of un-doped CdTe and Cl-doped CdTe devices were 0.45 nA mm^−2^ and 0.11 nA mm^−2^, respectively (it is already known from Fig. 3a that the leakage or OFF state current of Cl-doped device is lower than that of un-doped one). For next study, we conducted the modulation of X-ray beam exposure (R) using different beam times of 0.5, 1, 2, 4, 8 s at the same beam current (8 mA) and same peak voltage (90 kVp: peak acceleration voltage Vp is the maximum voltage for white X-ray generated by accelerated electron). With the increase of X-ray beam exposure, the signal level increases (the response signal was obtained by averaging the results from four turns of irradiation). For final sensitivity evaluation, the average photo-generated signal current from Cl-doped and un-doped devices was integrated with each beam time duration, which now becomes signal charges (Q_sig_). In Fig. 4d, such signal charges are plotted as a function of the exposure level (R) which is measured by X-ray dosage meter and appears linearly proportional to deposited energy: 90 kVp x 8 mA x time (sec). The dotted lines refer to the linear least-squares regression fit curves on the measured data, and hence their slopes indicate the X-ray sensitivities of sample detectors. The sample detectors almost linearly respond to the X-ray excitation (X-ray irradiation induced excitation). As a result, the sensitivity (25.74 nC mm^−2^ R^−1^) of Cl-doped CdTe Schottky diode appears approximately 50 times larger than that (0.48 nC mm^−2^ R^−1^) of the other device with un-doped CdTe. These sensitivity results are quite consistent with those from the photocurrent levels of Fig. 4a,b.

**Figure 5:**
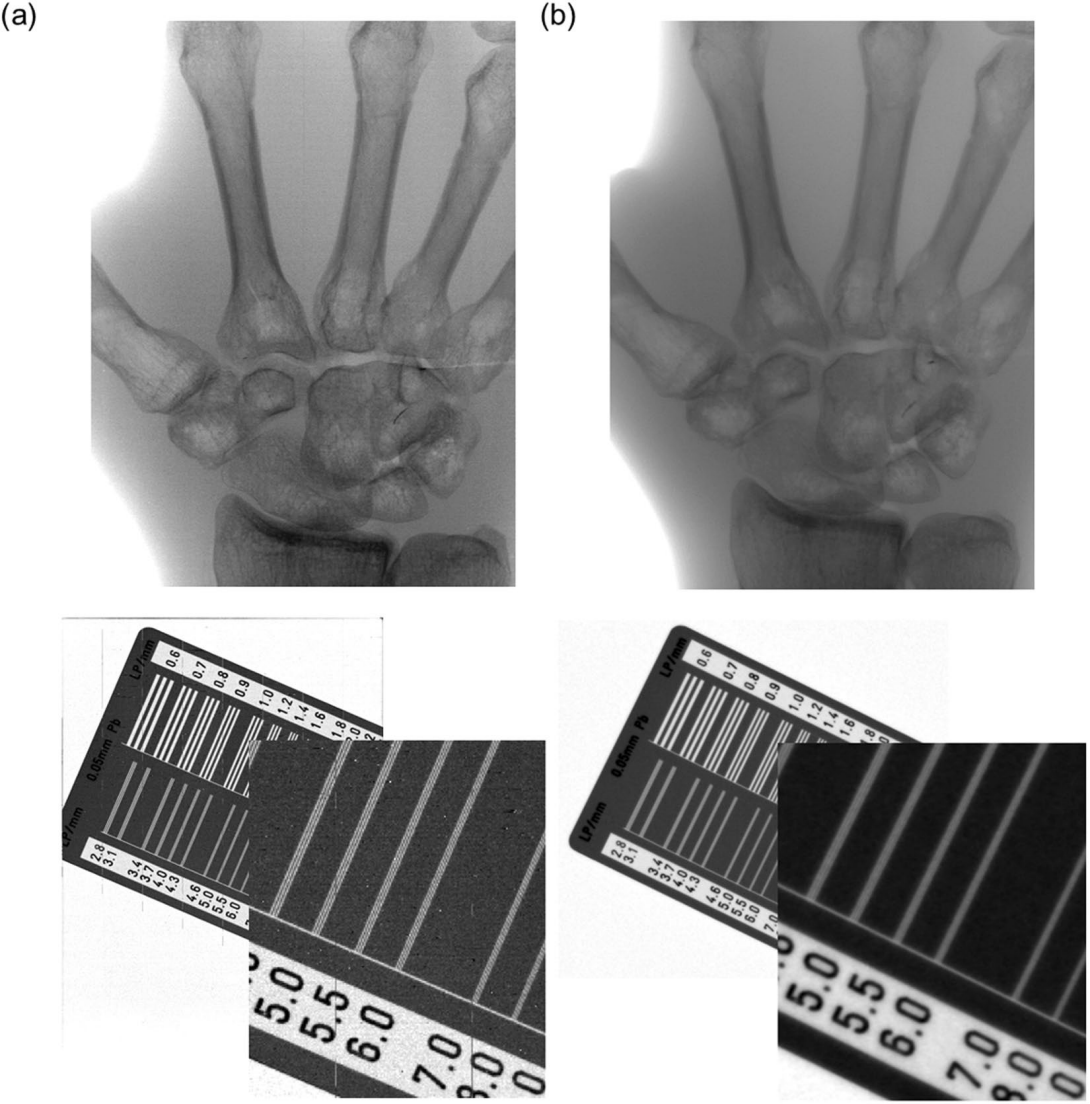
(**a**) Highly-resolved X-ray images of hand phantom and line chart as obtained from our direct conversion type X-ray APS system and (**b**) the same phantom and line chart images from the commercial indirect conversion type APS with CsI scintillator.

Inspired by aforementioned experimental results from the unpixelized blank Schottky diode with Cl doping, we finally fabricated 7 × 9 cm^2^ large area direct conversion type X-ray APS system, directly depositing the 150 μm-thick Cl-doped CdTe flm on CMOS structure as depicted in Fig. 1a. The imaging quality of our APS was compared with commercial indirect conversion type APS with CsI scintillator. Figure 5a,b display the images from our direct type APS and the indirect type commercial one, respectively. Those two images were obtained under the same X-ray energy and beam current condition (70 kVp and 6 mA). According to the figures of hand phantom and line chart^38^, the former is definitely clearer in image resolution than that of the latter which was obtained from indirect type detector. Such results were readily expected, however, the fabrication of directly grown 150 μm-thick CdTe detector on pixel patterned CMOS for large scale is world first, to the best of our limited knowledge. Moreover, 40 V low voltage operation for highly resolved X-ray image is also encouraging and worth recognition.

## Conclusion

In summary, we have fabricated direct conversion type CdTe Schottky diode for large scale X-ray detector based on CMOS pixel pattern APS system. We could for the first time deposit 150 μm-thick CdTe by thermal evaporation on large scale Ti/Al pixel pattern CMOS base at 400 °C, taking advantage of the minimal thermal expansion mismatch between Ti and CdTe. For low voltage operation, we applied Cl doping at all stage of CdTe film deposition to control or reduce the hole concentration of p-type CdTe, so that only 30–40 V reverse bias might be required to deplete the whole thickness of semiconducting p-type CdTe. Cl-doping appeared to cause both less textured grain structure and an order of magnitude lower leakage current in the Schottky diode than in the case without doping, enabling higher X-ray detection sensitivity. As a result, our CdTe Schottky diode/CMOS array demonstrates much higher resolution X-ray imaging in 7 × 9 cm^2^ large scale as a direct conversion APS imager than that of conventional CsI/CMOS array APS, an indirect conversion imager. To our limited knowledge, our results on polycrystalline CdTe Schottky diode/CMOS array would be very novel as a first demonstration of APS system equipped with directly deposited large scale X-ray detector.

## Experimental Section

A 150 μm-thick CdTe film was synthesized on CMOS substrates by using a multi-evaporation system as doped with around 150 ppm Cl using CdCl_2_ powder boat as shown in Fig. 1b. We controlled the initial vacuum level to be 9 × 10^−6^ torr at 200 °C and then during CdTe evaporation the process vacuum level was kept constant at a desired value in the range of 1~3 × 10^−5^ torr and at 400 °C. The thickness of the CdTe film and Cl doping rate were controlled by quartz thickness controllers and finally confirmed by cross section SEM images. After deposition process the films were annealed in a high vacuum ambient at 6 × 10^−6^ torr at 400 °C and then were cooled down to 150 °C at a very low cooling rate of 0.5 °C/min for preventing any possible thermal shock. All static and dynamic electrical measurements of our devices were performed with a semiconductor parameter analyser (Agilent 4155C) and in the dark at room temperature. C-V measurements on Schottky diodes were conducted with LCR meter (4284A, Agilent Technologies) at 1 MHz. X-ray dosage meter (Piranha R&F/M 605, RTI Electronics AB, Sweden) was used to initially measure total exposure of incident X-ray during the process of detection sensitivity measurement.

## Electronic supplementary material

Supplementary Information
