# Supplementary material for: Direct Thermal Growth of Large Scale Cl-doped CdTe Film for Low Voltage High Resolution X-ray Image Sensor
Source: Sci Rep. 2018 Oct 4;8:14810. doi: 10.1038/s41598-018-33240-1 (PMC6172199; doi:10.1038/s41598-018-33240-1)
Supplement: Supplementary file 1 — Supplementary Information [file 41598_2018_33240_MOESM1_ESM.docx]

Direct Thermal Growth of Large Scale Cl-doped CdTe Film for Low Voltage High Resolution X-ray Image Sensor

Silah Lee^1^, Jin Sung Kim^1^, Kyeong Rok Ko^1^, Gun Hwan Lee^2^, Dong Jin Lee^3^, Dong wook Kim^3^, Jin Eui Kim^3^, Ho Kyung Kim^4^, Dong Woon Kim^4^, & Seongil Im^1*^

Department of Physics and Institute of Physics and Applied Physics, Yonsei University, 50 Yonsei-ro, Seodaemun-gu, Seoul 120-749, Korea. ^2^Department Of Surface Technology, Korea Institute of Materials Science (KIMS), 797 Changwon-daero, Seongsan-gu, Changwon-si, Gyeongsangnam-do, 51508, Korea. ^3^Rayence Co. ,Ltd, 14, Samsung 1-ro 1-gil, Hwaseong-si, Gyeonggi-do, 18449, Korea. ^4^School of Mechanical Engineering, Pusan National University, 2, Busandaehak-ro 63beon-gil, Geumjeong-gu, Busan, 46241, Korea. Correspondence should be addressed to S.I. (semicon@yonsei.ac.kr)

**Supplementary Information**

Direct Thermal Growth of Large Scale Cl-doped CdTe Film for Low Voltage High Resolution X-ray Image Sensor

Silah Lee^1^, Jin Sung Kim^1^, Kyeong Rok Ko^1^, Gun Hwan Lee^2^, Dong Jin Lee^3^, Dong wook Kim^3^, Jin Eui Kim^3^, Ho Kyung Kim^4^, Dong Woon Kim^4^, & Seongil Im^1^

|  | **a-Se** | **c-Si** | **p-PbO** | **p-Cd(Zn)Te** | **p-HgI**_2_ | **p-PbI**_2_ | **p-TlBr** |
| --- | --- | --- | --- | --- | --- | --- | --- |
| ***Density* (g/cm^3^)** | 4.3 | 2.33 | 9.6 | 5.8 | 6.3 | 6 | 7.5 |
| ***δ* (μm)** | 976 | >10000 | 218 | 250 | 252 | 259 | 317 |
| ***E_g_* (eV)** | 2.2 | 1.12 | 1.9 | 1.7 | 2.1 | 2.3 | 2.7 |
| ***W* (eV)** | 45 | 3.62 | 8~20 | 5 | 5 | 5 | 6.5 |
| ***T_p_* (℃)** | 200 | 1414 | 100 | 600 | 100 | 200 |  |
| ***ρ* (Ωcm)** | 10^14^~10^15^ | 10^3^ | 10^13^ | 10^11^ | 4 x 10^13^ | 10^11^~10^12^ | 5 x 10^9^ |
| ***μτ* (cm^2^/V)** | 10^-6^~10^-5^ | 1 | 5 x 10^-7^ | 2 x 10^-4^ | 10^-5^~10^-4^ | 2 x 10^-6^ | 1.5 x 10^‑6^ |

**Table S1**. Electrical properties of photoconductive sensor materials

*Note from the table; a-, c-, and p- respectively indicate amorphous, single crystalline, and polycrystalline. ***δ*** means X-ray absorption depth at 60 keV while ***T_p_*** does process temperature. ***E_g_*** is energy band gap. ***W*** means electron-hole pair creation energy and ***μτ*** signifies mobility × carrier life time product. A low process temperature signifies some possibility of materials instability. [Courtesy from S. O. Kasap, M. Z. Kabir, J. A. Rowlands, Curr. Appl. Phys. 2006, 6, 288.].


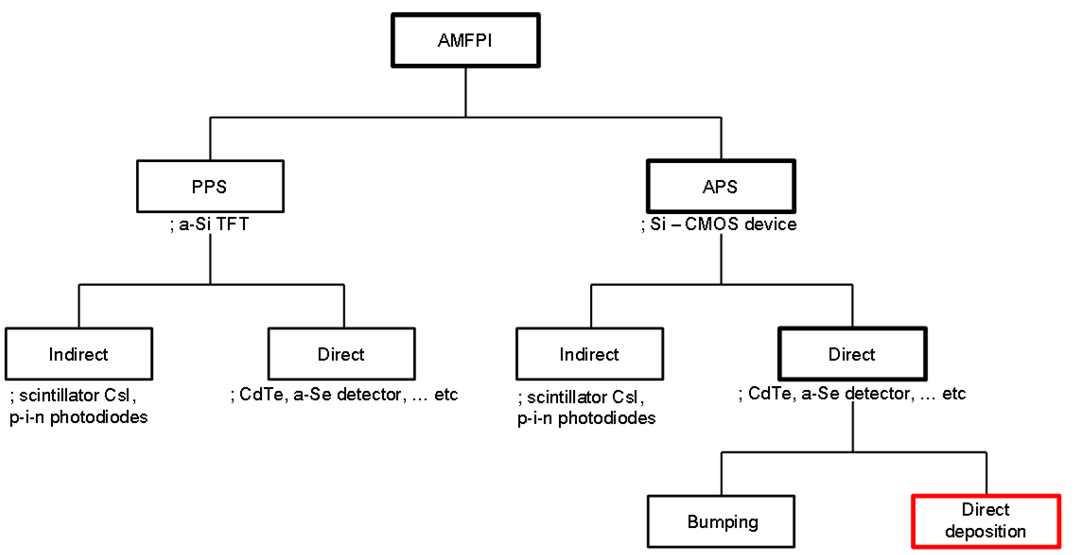


**Figure S1.** Summary of all active matrix flat panel (pixel) imager (AMFPI) which is categorized into passive pixel sensor (PPS-using thin film transistor: TFT) and active pixel sensor (APS-using CMOS) as back panel of X-ray imaging system. Those image sensor systems (PPS and APS) can use indirect or direct front panel X-ray detector, while direct type detector is prepared by bumping or direct deposition. The last highlighted box indicates our choice of APS with direct deposition.


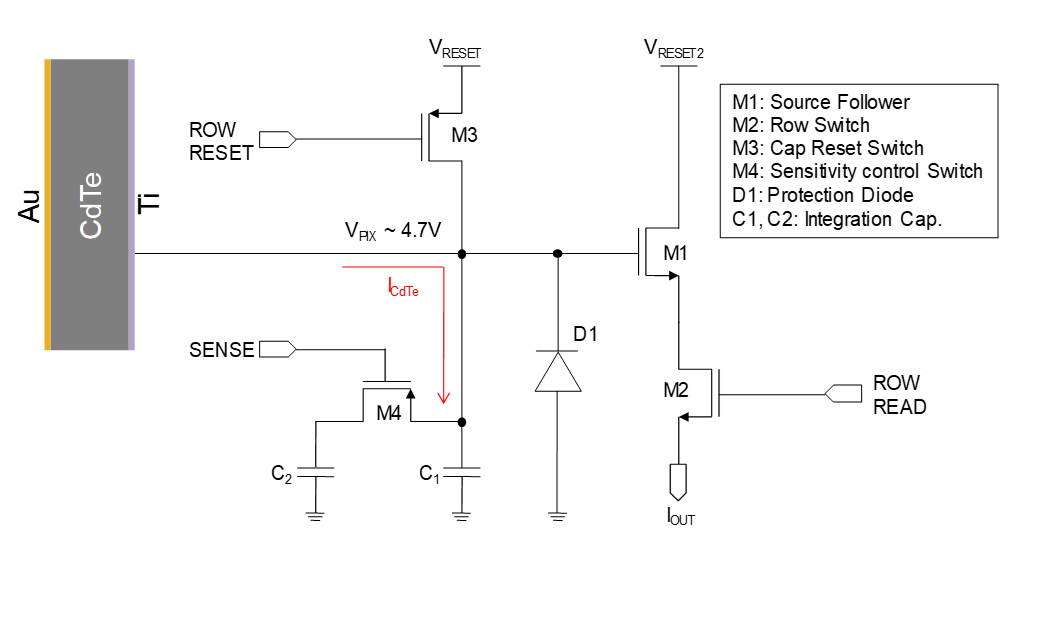


**Figure S2.** Details of CMOS readout circuits to support our APS system
